# Quantum Dots for Molecular Diagnostics of Tumors 

**Published:** 2011

**Authors:** T.A. Zdobnova, E.N. Lebedenko, S.М. Deyev

**Affiliations:** Shemyakin and Ovchinnikov Institute of Bioorganic Chemistry, Russian Academy of Sciences

**Keywords:** quantum dots, fluorescence imaging, multicolor labeling, nanoparticles

## Abstract

Semiconductor quantum dots (QDs) are a new class of fluorophores with unique physical and chemical properties, which allow to appreciably expand the possibilities for the current methods of fluorescent imaging and optical diagnostics. Here we discuss the prospects of QD application for molecular diagnostics of tumors ranging from cancer-specific marker detection on microplates to non-invasive tumor imaging*in vivo*. We also point out the essential problems that require resolution in order to clinically promote QD, and we indicate innovative approaches to oncology which are implementable using QD.

##  Introduction 

 In recent biomedical studies, much attention has been paid to the search for new methods of noninvasive imaging of the internal structure of biological objects. Instruments with a high spatial resolution have been designed, and, consequently, optical methods for investigation are gaining widespread use. One of the most demonstrable and informative methods among these is the fluorescent diagnostics of pathological foci directly in the organism. 

 A considerable portion of the methods being developed are directed toward imaging tumors, tissues and organs; studying the molecular structure of tumor cells by auto-fluorescence registration; or by specific staining of the objects under observation with fluorescent contrasting dyes. Methods such as these enable us not only to localize a tumor in the organism, but also to estimate the level of expression of various proteins, as well as the activity of individual cells and the processes that have an impact on tumor behavior and its response to the action of therapeutic agents. 

 In modern methods of diagnostics, special demands are placed on the contrast agents used. Fluorophores must possess the following properties: small dimensions (1–10 nm); sufficient brightness and a high quantum yield; it is necessary that their excitation and fluorescence in the spectral range correspond to optimum penetration into biological tissues; and chemical stability, photostability, and biocompatibility (stability in biological media and nontoxicity). Moreover, frequently in order to perform biological studies, these fluorophores need to be conjugated with different targeting molecules, so that they can be delivered to particular targets (proteins, compartments, and cells). The conjugates need to specifically interact with the target and do so in a stable manner, whilst possessing a low level of nonspecific binding. 

Fluorescent semiconductor nanocrystals, so-called quantum dots (QDs), are a relatively novel class of fluorophores with unique optical and physicochemical properties, atypical of other fluorescent dyes. Two major classes of fluorophores have been conventionally used for diagnostics: organic dyes and fluorescent proteins [1]. These fluorophores were first used in biology and medicine and have subsequently evolved significantly. At this point, a large variety of organic dyes with a small molecular weight and fluorescent proteins, characterized by high brightness, good quantum yield and emission over the entire spectral region from blue to the near-infrared (IR) region, have been designed [2, 3]. However, some of the properties of these fluorophores (in particular, broad emission spectrum and low photobleaching thresholds) still limit their effectiveness in such types of studies as long-term imaging and ‘multiplexing’ (simultaneous detection of multiple signals) without an additional complex of instrumentation and processing [4].

QDs possess a number of physicochemical features that open wider possibilities in comparison with conventionally used fluorescent labels, making them particularly attractive for use in various biological experiments [5, 6].

**Fig. 1:**
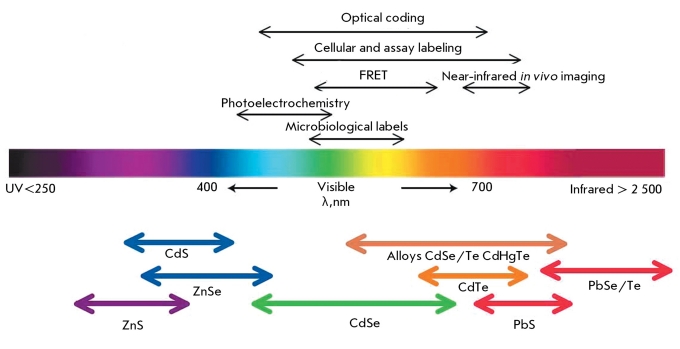
Representative QD core materials scaled as a function of their emission wavelength superimposed over the spectrum. Representative areas of biological interest are also presented corresponding to the pertinent emission, highlighting how most biological usage falls in the visible – near-infrared region. Reprinted by permission from Macmillan Publishers Ltd.: [Nature Materials] (Medintz I.L., Uyeda H.T., Goldman E.R., Mattoussi H. Nat Mater. 2005 4:435-446), copyright (2005).

This review will summarize how QDs can be used for studying the molecular mechanisms of the processes that occur in tumor cells and for both *in vitro* and *in vivo* tumor diagnostics.

##  1. QD FEATURES PROVIDING ADVANTAGES WHEN USING THEM IN BIOMEDICAL STUDIES 

Quantum dots are almost spherical nanocrystals 1–10 nm in diameter, consisting of a small number of atoms (500–10,000) of semiconductor materials of groups II–VI (e.g., CdSe, CdTe, CdS, and ZnSe) or groups III–V (e.g., InP and InAs) of Mendeleev’s periodic table. The term “dot” mainly characterizes the extremely small dimension of these objects; while the adjective “quantum” describes the fact that their behavior and properties are described to a significant extent by quantum mechanics, rather than classical mechanics. The decrease in the particle size of the compound to a value smaller than the exciton Bohr radius (e.g., for spherical СdSe particles this diameter is less than 6 nm) results in that the properties of the compound are determined not as much by their chemical composition as by their particle size. In light of this, semiconductor nanocrystals are characterized by their unique optical characteristics and physicochemical properties that distinguish them favorably from other fluorophores that are conventionally used in biology [7].

QDs possess a high molar extinction coefficient (higher than that of organic dyes by a factor of 10–100) and a high quantum yield (up to 90%), which provides to these fluorophores exceptional brightness. QDs are characterized by a broad absorption spectrum, a considerable Stokes shift, and a narrow and symmetrical (without a “tail” in the red region) fluorescence spectrum (peak width ~25–40 nm). In this regard, the emission wavelength is core size-tunable, enabling us to create a wide range of various QDs fluorescing within a spectral range from UV to IR (400–2,000 nm), using the same materials and the same procedures ( *Fig. 1* ). Furthermore, the broad excitation spectrum typical of these nanoparticles (QDs can be excited by light at any wavelength smaller than their fluorescence wavelength) allows to excite a mixture of different QDs at one wavelength that is considerably remote (> 100 nm) from their fluorescence wavelengths [9]. Such properties of QDs significantly increase their potential use in multicolor labeling and the simultaneous identification of different biological objects, in comparison with other dyes [6].

High resistance to photobleaching (that is higher than that of organic fluorophores by a factor of 100–1,000) and an exceptional stability towards photo- and chemical degradation [7, 10], which is typical of fluorescent semiconductor nanocrystals, makes it possible for us to use them in long-term experiments on real-time imaging of the processes occurring inside a cell (e.g., endocytosis) [11] or translocation of individual receptor molecules along the living cellular surface and for staining the samples that require long-term storage [13].

A more detailed description of the physicochemical properties of QDs important for their biological application, and a comparative evaluation of their use and that of other fluorophores in biomedical studies, can be found in reviews [4–6].

Physicochemical and optical properties and the features of QDs directly depend on the method of their synthesis. This wide field (not an issue for this review) is still under development. It is increasing the number of QDs used in biomedical studies and enhancing their properties (ref. reviews [4, 5]).

 Until recently, two types of water-soluble monodisperse QDs had been in use in biology: the so-called bioinert nanocrystals and nanocrystals conjugated to various biological molecules in order to add certain specificity to them. 

Bioinert QDs find application as nonspecific contrast agents for cell staining due to endocytosis, for the contrasting of blood vessels and lymph nodes, and for studying biodistribution, toxicity, and *in vivo* passive delivery of nanoparticles into animal tumors. Water-soluble QDs modified with hydrophilic thiols [14] and encapsulated by silicon or amphiphilic polymers [16] are frequently used as such bioinert particles. Such particles are typically coated with a layer of inert molecules, in order to reduce the nonspecific binding; the manufacturers of commercial QDs usually use polyethylene glycol (PEG) for this purpose.

##  2. TARGETING OF QDs TO TUMOR CELLS 

 It is a common requirement when using QDs as fluorophores for tumor imaging that they bind to various targeting molecules, thus ensuring the selective delivery of QDs to tumor cells and their components. The specificity of labeling is provided by the selection of a target that optimally suits each particular case and the corresponding targeting molecule. 

The receptor part of signal proteins that are overexpressed on tumor cell membranes is used most often as a specific target. The level of expression of these cellular molecular oncomarkers, determined directly in the tumor tissue, characterizes the molecular profile of each individual tumor and is used to determine the immune status of the tumor and the individualization of therapeutic treatment [17].

 Antibodies and their fragments, ligands of specific receptors localized on the tumor cell surface, small molecules (such as peptides and aptamers) with specific affinity for some of the oncomarkers are used as a targeting module, which provides the selective delivery of QDs to tumor cells and their components, depending on the aims and objects of the study. 

**2.1 Targeting agents**

Immynoglobulin (Ig) molecules have been known for a long time, as they are widely used as efficient targeting modules for the specific delivery of diagnostic and therapeutic agents both *in vitro * at the cell and tissue level and *in vivo * at the whole body level. As early as in one of the first studies devoted to using QDs for biological investigations, the potential to obtain complexes of QDs with IgG molecules and the ability of the resulting complexes to bind to specific antispecies polyclonal antibodies and to form precipitates in the solution were illustrated [14]. Later, such complexes were used for the labeling of particular molecules located in various cell compartments (on the membrane surface, in cytoplasm, and in the cellular nucleus) [16].

Regardless of the wide distribution of full-size antibodies in diagnostic systems *in vitro* , their application as targeting agents *in vivo * usually requires the elimination of their effector functions and a radical modification of physicochemical properties [18]. Antibodies of scFv format are those that best meet these requirements [19, 20]. These small antibody fragments do not contain a constant domain. Although this fact has no effect on their targeting properties, it reduces the possibility of the side effects caused by the interaction between the constant domains and receptors of the cells of the immune system and proteins of the complement system [21]. scFv antibodies against surface oncomarkers are widely used as targeting modules for the fluorescent imaging of tumor cells and delivery of therapeutic agents to them [22–24].

At the Nie laboratory, it was demonstrated that QDs conjugated to targeting mini antibodies scFv could be used for tumor imaging, including *in vivo * imaging. Accumulation by tumor cells and efficient internalization of QDs conjugated to a human anti-EGFR-antibody of the scFv format were observed after their intravenous injection into a human pancreatic bearing mouse [25].

The major bottleneck of scFv antibodies as targeting agents is their monovalence, since monovalent binding to an antigen on the cell surface does not ensure the long-term retention of the antibody and results in its rapid dissociation [26, 27]. Meanwhile, the typically large surface area of QDs makes it possible to attach several scFv molecules to each nanoparticle and create unique multivalent constructions with enhanced properties [28].

**Fig. 2:**
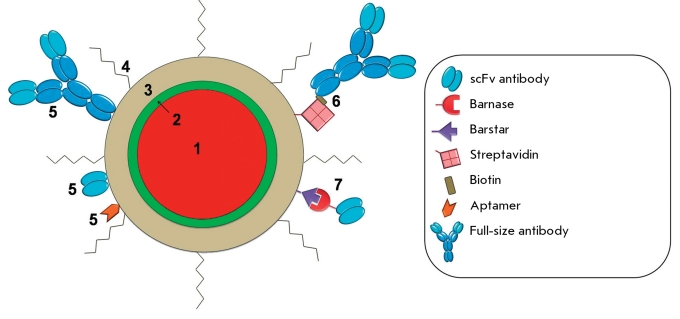
Design of a current quantum dot for biomedical application. (1) –fluorescent core (usually CdSe or CdTe); (2) - protective shell (usually ZnS); (3) – polymer coating to provide colloidal stability, and direct linkage to biologically active molecules, (4) – PEG, (5) – targeting molecules joined with QD directly or through biotin-streptavidin (6) and barnase-barstar (7) adaptor systems.

Peptides are used as targeting molecules in order to perform specific recognition of certain proteins, with the purpose of imaging cells and their components [29]. The application of this approach in the design of targeted QDs was first demonstrated for short recombinant peptides that were capable of specific recognition of integrin in human neiroblastoma studies [30]. Later, it was proven that this approach could also be used in the specific labeling of the cells of lung endothelium, brain endothelium, and human breast carcinoma both *in vitro* and in living cells [31]. Arginyl-glycyl-aspartic acid (RGD peptide) capable of recognizing integrin has been noted as a good alternative targeting agent for the fluorescing in the IR range QDs during *in vivo* imaging of different tumors in the mouse organism [32].

Another promising targeting agent for the delivery of QDs to tumor cells is aptamers; the specially designed oligonucleotides capable of recognizing certain proteins and cell components and binding to them with high specificity. Various aptamer-based conjugates have been successfully used for cell imaging and recognition, biomarker detection, etc. [33]. Conjugates of QDs with the aptamer specific to the PSMA cancer marker selectively stained immobilized and living prostate tumor LNCaP cells and the same cells in a model medium of collagen matrix [34]. It was shown that the use of aptamers as a targeting agent for imaging prostate tumor cells using QDs was equally efficient as using QDs conjugated to anti-PSMA-antibodies, but considerably less expensive [35]. QD-aptamer conjugates can be used in parallel with other targeting agents, such as peptides, for the simultaneous imaging of several oncomarkers [36]. Moreover, the preparation of biotin-conjugated aptamers, which have the ability to bind to any streptavidin-conjugated QDs, provides the possibility to create universal reagents for the two-stage delivery of QDs to tumor cells [33].

**2.2 Methods for binding targeting agents to QDs**

In contemporary practice, two major approaches to binding the targeting molecules to QDs are used: direct binding (typically covalent) of protein molecules to active groups on the QD surface and adaptor-mediated binding ( *Fig. 2* ).

Usually, protein molecules are bound directly to the semiconductor part of a nanocrystal (via the SH group or by metal-affine coordination of histidine residues with the zinc atoms of a nanocrystal shell) or to its hydrophilic coating (by conjugation with carboxyl, amine, and thiol groups using special catalysts; via electrostatic interaction). These methods were thoroughly described in the reviews [5, 37].

The surface area of a nanocrystal is appreciably large and is accessible for the binding of several biological molecules. A range of 2 to 5 protein molecules and more than 50 small molecules (oligonucleotides or peptides) can be bound to one nanoparticle 4 nm in diameter [38]. It should be noted that the reactivity of certain types of biological molecules after direct conjugation with nanocrystals can vary considerably. In particular, although antibodies retain their specificity after conjugation, they lose their affinity considerably [39]. Moreover, direct conjugation of QDs with antibodies requires that the antibody activity in each new conjugate be checked.

 The use of so-called “self-assembling adaptors” (small adhesive molecules that bind to each other with high efficiency and specificity but do not form homodimers) is a more promising approach to QD binding to antibodies. The formation of complexes with these small molecules has no considerable effect on antibody affinity and allows us to simply prepare various combinations of antibodies with different specificities to QDs that fluoresce in different ranges, without any additional modifications. Heterodimerization modules that were previously designed for the preparation of recombinant bispecific and multivalent antibodies and for two-stage delivery of therapeutic agents to the tumor are used as adaptor molecules to bind QDs to antibodies. 

The streptavidin–biotin system is the most well-known and broadly used system among these modules; it possesses a high binding affinity *K*_a_ ~ 10 ^-14^ – 10 ^-15^ М [40]. Streptavidin is attached to QDs covalently or via electrostatic interactions, which allows them to bind to biotin-conjugated targeting agents. Streptavidin-conjugated QDs were first used for imaging of the tumor marker HER2/neu on the surface of human breast tumor SKBR-3 cells through biotin-conjugated anti-human secondary antibodies and humanized anti-HER2/neu antibodies [16]. A similar three-stage system was used for binding QDs to antibody fragments specific to glycyn receptors on a neuron membrane, making it possible to observe the motion of individual receptors in living neurons [41]. The three-stage system based on the biotin–streptavidin affine pair (primary antibodies; biotinilated secondary antibodies; streptavidin-conjugated quantum dots) allows for the use of single streptavidin-conjugated quantum dots for the imaging of a number of various targets without any additional modification, since the labeling specificity is determined by the corresponding primary and biotinilated secondary antibodies.

By using primary antibodies that bind to QDs via a biotin–streptavidin bridge [11], the number of stages in the labeling process can be reduced to two. This approach is not only used for antibodies, but also for many other targeting agents. Thus, QDs conjugated with an integrin-recognizing peptide via a biotine–streptavidin module have been successfully used for labeling the αν-subunit of integrin in human neuroblastoma cells SK-N-SH [27].

Because of its universal nature, the streptavidin–biotin system is now widely used in certain types of immune diagnostic investigations that use QDs. Streptavidin-conjugated QDs and biotin-conjugated antibodies became commercially available recently (e.g., see www.invitrogen.com). However, it is important to note that the application of this system to create site-directed fluorophores for tumor imaging in a human organism *in vivo * is restricted by the presence of a large quantity of endogenous biotin, which can compete with biotinilated components, thus reducing the labeling efficiency.

In order to prepare antibody-conjugated QDs, we propose the use of a barnase–barstar adaptor module, which has shown good results in the preparation of heterodimeric mini-antibodies and their fluorescent derivatives [26, 42, 43]. This adaptor module is based on the ability of ribonuclease barnase from *Bacillus amyloliquefaciens * to form a very stable complex *(Kd* ~ 10 ^-14^ М) with its natural protein inhibitor, barstar [26].

Since the binding regions of barnase and barstar are localized outside their N- and C-terminal parts, each of these proteins is accessible for fusion with scFv antibody fragments. Meanwhile, the binding efficiency of module components is retained. The small dimensions of barnase and barstar (110 and 89 a.a., respectively), stability, good solubility, and stability towards proteases allow to produce appreciable quantities of the desired chimeric proteins in bacterial producers. Moreover, barnase, within recombinant proteins, serves as an intramolecular chaperone, ensuring the correct folding of recombinant proteins, which is particularly important when designing structures with targeting antibodies [44].

**Fig. 3:**
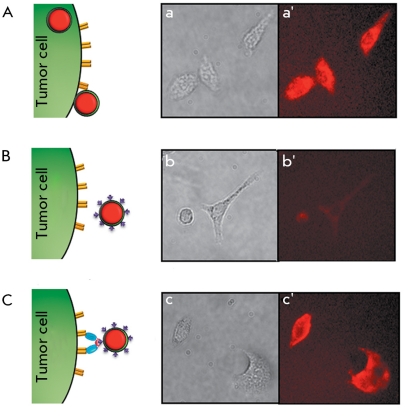
Non-specific and specific interaction of QDs with tumor cells. Schematics (on the left) and results (on the right) of fluorescent microscopy of SKOV-3 cells after incubation with QD (А), with QD-barstar (B), and with anti-HER-2/neu scFv dimer joined with barnase, following QD-barstar (C). Cell images in visible light (a, b, c) and fluorescent cell images (a’, b’, c’) are shown. Legend see *Fig. 2* .

The small dimensions and extreme stability over a wide range of conditions make it possible to easily form conjugates of both barnase and barstar with active groups on the QD surface. It was also found that the conjugation of QDs with barstar considerably reduces the non-specific binding of QDs to the cellular membrane. Hence, fluorescent nanocrystals, which usually adhere nonspecifically to human ovarian adenocarcinoma SKOV-3 cells and penetrate them ( *Fig. 3A* ), become virtually neutral with respect to these cells after conjugation with barstar ( *Fig. 3B* ). At the same time, the barstar located on the QD surface provides the additional binding of targeting antibodies using the barnase–barstar adaptor system, ensuring the efficient and specific labeling of cancer cells ( *Fig. 3C* ).

 An important advantage of the barnase–barstar module is the accurate (1 : 1) ratio between the components in the complex and the total absence of self-aggregation, as well as a high interaction affinity, higher than that of all other dimerization systems, with the exception of the streptavidin–biotin system. As opposed to the streptavidin–biotin system, the use of the barnase–barstar system is based on genetic engineering technologies and requires no covalent modifications of antibodies. 

The barnase–barstar adaptor system has been successfully used for the preparation of fluorescent complexes for the imaging of tumor cells overexpressing oncomarker HER2/neu, based on 4D5scFv antibodies and QDs of two types 1) QDs modified by mercaptoacetic acid and 2) QDs covered with a polymeric shell [45, 46]. In both of these cases, efficient and selective staining of membranes after the incubation of breast adenocarcinoma cells and human ovarian adenocarcinoma cells with the obtained fluorescent complex was observed.

**Fig. 4:**
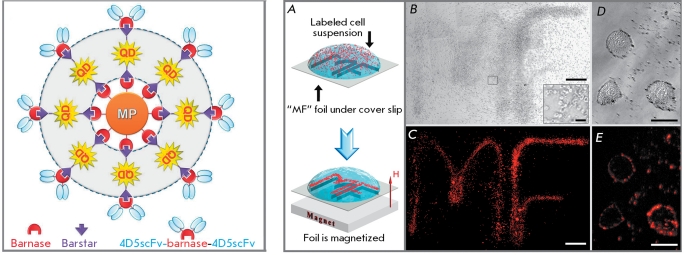
Superstructures consisting of QDs, magnetic particles (MP), and scFv antibodies. Conceptual design of superstructures based on barnase-barstar adaptor system (on the left), and multifunctionality proving (on the right). Human ovarian cancer SKOV-3 cells labeled by the assembled trifunctional structures were dragged toward the contour of letters “MF” (A). Bright-field ( *B* - 100x magnification and D - individual cells) and fluorescent (C - 100x magnification and E - individual cells) photos of the sample. Adapted by permission from the National Academy of Sciences of the United States of America: [Proc. Natl. Acad. Sci. USA] (Nikitin M.P., Zdobnova T.A., Lukash S.V., Stremovskiy O.A., Deyev S.M. Proc Natl Acad Sci USA. 2010 107:5827-5832), copyright (2010).

Furthermore, it was shown that QDs conjugated to targeting antibodies, with the help of adaptors, can be bound to molecules or nanoparticles of a different nature. Thus, they can be regarded as components of a “Molecular Lego kit” [43]. By implementing the conception of such a Lego kit, self-assembling multimodal structures were designed on the basis of the barnase–barstar adaptor module using QDs and magnetic particles. The resulting fluorescent magnetic nanoparticles are supplied with humanized mini-antibodies against the HER2/neu oncomarker and can efficiently and selectively label the corresponding tumor cells [28]. As a result, fluorescence-labeled tumor cells acquire responsiveness to a magnetic field ( *Fig. 4* ).

**2.3 The problem of nonspecific binding of QDs**

QD tendency to “adhesion,” i.e., nonspecific binding to the cellular membrane, proteins, and components of the extracellular matrix, and their uncontrolled penetration into cells is a significant impediment to selective QD-based fluorescence labeling of biological objects. For example, particles with a strong negative or positive charge containing on their surface carboxyl or amino groups, respectively, were shown to possess a high level of nonspecific binding with cells and tissues [47, 48]. Such nonspecific binding can be explained by the electrostatic interaction between charged groups on the QD surface and the charged regions of proteins and other molecules on the cellular surface.

An additional explanation for the nonspecific binding of QDs with the cellular surface may be the hydrophobic interaction between the lipids in the cellular membrane and the stabilizing agent molecules (e.g., tri- *n* -octylphosphine or oleate anion) that remain on the QD surface after their synthesis due to the incomplete coating of a nanoparticle core with a ligand providing hydrophility, or due to instable binding of this ligand. Thus, it was shown that QDs comprising cysteine, MAA, dihydrolipoic, and other mercapto-carboxylic acids, notable for their dynamic and instable Zn–S bond, exhibit the highest level of nonspecific binding [47].

It was determined that the degree of nonspecific binding strongly depends on the cell type [47, 49], which can be explained by different contents of charged and hydrophobic regions on the membrane of particular cells.

In order to reduce the degree of nonspecific binding, QDs are additionally coated with a layer of inert molecules. One such substances, widely used now, is PEG, a nontoxic hydrophilic polymer commonly used for enhancing the biocompatibility of drugs [48]. Quantum dots modified by PEG have a surface charge that is close to neutral and remain colloidally stable under various experimental conditions. Furthermore, PEG reduces the ability of QDs to interact with the cell surface or with the proteins of an extracellular matrix; i.e., it results in the passivation of the QD surface [50].

In the case of PEG-coated QDs, it should be noted that despite the fact that they are successfully used both *in vitro * and *in vivo * experiments, such a modification is not sufficient for some other purposes. Furthermore, PEG-coated particles have a considerably higher hydrodynamic diameter, which impedes their access to biological targets [51].

In order to minimize the nonspecific binding of QDs without increasing their size, a method for coating nanocrystals with a neutral hydroxyl layer was proposed [52]. The hydrodynamic diameter of the resulting nanocrystals is 13–14 nm, which is smaller than the size of PEG-modified nanocrystals by 50%. When using the obtained complexes for HeLa cells imaging, 140-fold and 20-fold reductions of nonspecific binding, in comparison with carboxylated QDs and biotin-conjugated QDs, respectively, were observed. To perform the targeted delivery of such nanocrystals, it is necessary to supply them with targeting molecules, resulting in a partial loss of material and a decrease in the yield of the final product. Meanwhile, it is quite realistic to reduce the nonspecific adhesion of QDs on the cellular membrane and simultaneously provide nonspecific binding of a nanoparticle to certain receptors expressed on the surface of a tumor cell. It was noted that some small neutral molecules, such as peptides or small proteins, enable to reduce the nonspecific binding of QDs [52]. We demonstrated that the component of the adaptor system, barstar, also possesses this property (see above Section 2.2, *Fig. 3* ).

## 3. *In vitro * Diagnostics

One of the most promising and rapidly developing areas of application of QDs is their usage as fluorescent labels during *in vitro* study of tumor cells: for imaging tumor cells and for localizing the individual molecules expressed in them. The unique properties of QDs, which make it possible to perform multicolor labeling and long-term observation of fluorescence of objects, allow one to considerably broaden the range of conventional methods that are used in this field. *In vitro* diagnostics is now the only application of QDs out of all alternatives of the biomedical use of QDs which can be quickly implemented in clinical practice (as opposed to the *in vivo* use of QDs, which requires long investigations of QD toxicity and further consequences of their introduction into the organism).

 The major directions of investigation include: 1) imaging of tumor cells overexpressing certain oncomarkers, 2) staining of tissues and their sections; and 3) observation of individual molecules and cells in real time. 

**3.1 Imaging of tumor cells**

The imaging of tumor cells and identification of the individual oncomarkers within them is of great practical importance. Most of the oncomarkers used for imaging are represented by receptors overexpressed on the membrane surface of tumor cells, and they are almost non-expressed in normal tissues. A high level of expression of such markers correlates with a tumor process in the organism; their detection and quantitative assessment being important for the early diagnostics, classification, and therapy of tumors [51].

Several years after pioneering studies on the design and use of biocompatible QDs were published [14, 15], a few research groups claimed that it was possible to use QD conjugates for the imaging of tumor cells. Thus far, QDs conjugated to various targeting agents (antibodies, ligands, peptides) have been known. They are intended for visualizing the cells of clinically significant human tumors: prostate carcinoma [53], breast adenocarcinoma and ductal carcinoma [21, 54, 55], pancreatic carcinoma [56], glioblastoma [32], and squamous cell carcinoma of the tongue [57].

Efforts in this area have primarily focused on the optimization of the properties of QDs for their application in experiments *in vivo* and at the whole-body level. The preliminary purpose was a good solubility in aqueous solutions, biocompatibility, low toxicity of QDs, and additionally their supplying with targeting molecules providing the specificity of labeling. Over a short period of time, appreciably simple, inexpensive, and well reproducible QD-based methods for the imaging of cancer cells were designed for the diagnostics of clinically significant tumor types and prognosis of the disease’s progresson ( *Table* ). Hence, significant methodical groundwork was laid for implementing these methods in clinical practice and further *in vivo* studies aimed at the imaging of tumors and their metastases directly in the living organism.

**3.2 Simultaneous detection of several oncomarkers**

Generally, targeting molecules (antigens, peptides, aptamers, etc.) that selectively bind to the surface oncomarker provide a high specificity of labeling of the corresponding tumor cells [17]. At the same time, such a feature of tumor cells as their extreme variability during the development of the disease and response to the action of therapeutic agents raises for researchers the problem of simultaneous imaging of several surface markers (see Section 1).

The fundamental possibility of using QDs for simultaneous multiplex detection was demonstrated on five tumor markers in a human breast tumor cell culture. The simultaneous detection of the receptors ER, PR, EGFR, mTOP, and HER2/neu using QDs fluorescing in different spectrum regions correlates positively with the results of conventional methods; including immunohistochemistry, western blotting, and fluorescence *in situ* hybridization, while it considerably increases the rate of analysis and reduces cost [61].

Simultaneous imaging of two hypothesized cancer markers - integrin α _v_ ß _3 _ and nucleolin-using QDs conjugated to the RGD peptide and aptamer AS1411, respectively, enables to compare the localization of these markers in the cell [36]. Internalization of nucleolin and the surface distribution of integrin were confirmed using confocal microscopy, which will probably allow to better understand how they participate in the processes occurring in tumor cells.

 The results of these studies demonstrate that QDs conjugated to targeting molecules have a powerful potential as components of novel systems for assessing tumor types, their progression stage, and the metastatic potential on the basis of multiplex imaging. 

Fundamental studies of oncological processes, in addition to the detection of the markers that are overexpressed in cancer cells, require that a number of other proteins, frequently characterized by a low-copy number, be revealed. The golden standard today for identifying low-copy number proteins is enzyme-linked immunosorbent assay (ELISA); its sensitivity attains the picomolar value. This method has been widely used; however, it is quite labor-intensive, expensive, time-consuming, and does not allow for multiplexing. The replacement of organic fluorophores and colorimetric reagents in immune enzyme studies by QDs alone does not provide a significant advantage in terms of sensitivity (the sensitivity of the analysis with QDs is ~ 100 pmol) [70]. It is the application of QDs with different spectral characteristics which allows simultaneous detection of several proteins to be performed. Thus, four toxins were simultaneously detected using four different QDs, which emitted between 510 and 610 nm, in a sandwich immunoassay configuration with a single extitation source [70]. Unfortunately, these authors have not managed to carry out the quantitative assessment at this stage; further investigations are required to design a good immunofluorescence test. Another study demonstrated the simplicity and obviousness of the simultaneous detection of two proteins with two spectrally different QDs in a western blot assay [71]. Unquestionably, simultaneous multicolor labeling using QDs is a novel and powerful method that will allow us to solve both conventional and fundamentally new problems that previously could not be solved or were extremely labor-intensive when conventional approaches were used.

**Fig. 5:**
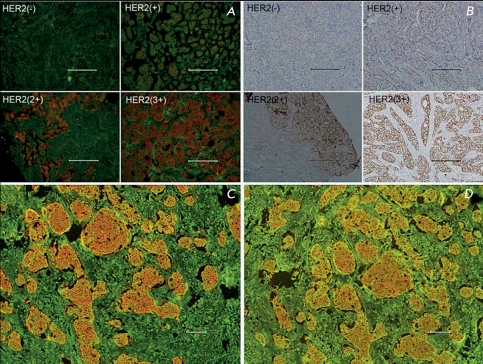
Advantages of QDs for immunohistochemical assays. A – Specimens with different HER2 IHC scores detected by QD-IHC (A) and by conventional IHC using peroxidase (B). Preservation of QD fluorescence and photobleaching on day 2 (C) and day 75 (D). *Scale bar* 100 µm. Adapted by permission from Elsevier: [Biomaterials] (Chen C, Peng J, Xia H, Yang G, Wu Q, Chen L, Zeng L, Zhang Z, Pang D, Li Y. Biomaterials. 2009 30:2912-2918), copyright (2009).

Simultaneous multiplex labeling using QDs with different spectral characteristics also provides indisputable advantages in studies requiring high-performance screening of molecules. QDs are successfully used for the analysis of various components of cell systems using microarray technology and for the parallel analysis of the genome and proteome content of healthy and affected cells [72]. The brightness and stability of QDs significantly increase the sensitivity and the possibility of parallel detection of the components of complex mixtures. The results obtained can help better understand the signal paths in cells, as well as be used in the design of new therapeutic approaches.

**3.3 Immunohistochemical assay**

 Immunohistochemical assay (IHC) is the method for tumor diagnostics most widely used in clinical practice. This method of morphological study is based on imaging and microscopic evaluation of the results of the antigen–antibody reaction in biopsied tissue sections and allows not only to detect the presence and intensity of a signal, but also to evaluate signal distribution over the cell (staining of the membrane, cytoplasm, nucleus, and other structural elements). Immunochemical staining of formaldehyde-fixed and paraffin-embedded tissue sections of tumor biopsy samples is a complicated task because of high tissue autofluorescence and the reduction of the amount of antigen during the fixation and paraffin embedding. 

QDs appeared very well-suited for the resolution of this problem. Images of fixed sections of human skin basal carcinoma [60], mouse breast tumor overexpressing the human receptor HER2/neu [16], and basal-squamous cell carcinoma of human skin [73] were obtained using QDs. It was also demonstrated by the example of the human breast tumor that QD-based probes can be designed for quantitative and highly sensitive detection of the low expression of cancer surface markers, in particular, oncomarker HER2/neu [74]. Researchers have also noted the excellent photostability of QD-stained samples: their fluorescence intensity remains intact for 9–75 days [74] ( *Fig. 5* ).

The combination of conventional IHC procedures with QD-based fluorescent dyes allows one to considerably improve the resolution and sensitivity of the method (see review [17]) and provides a possibility of simultaneous imaging of several markers [75]. Moreover, the application of QDs makes the IHC method much more illustrative ( *Fig. 5* ).

**3.4 Real-time detection of molecular processes and cells**

The high resistance of QDs to photobleaching and their high level of brightness enable us to use them for imaging of the processes occurring in cells, including tracing the dynamics of individual molecules [41]. Certain membrane proteins are of special interest; the investigation of their localization and dynamics is crucial in understanding such processes as chemotaxis and inter- and intracellular signal transduction. Thus, QDs conjugated with the corresponding targeting ligands have been successfully used for imaging of the dynamics of the receptors of glycine [12] and γ-amino butyric acid [76] in a neuron cell culture.

**Fig. 6:**
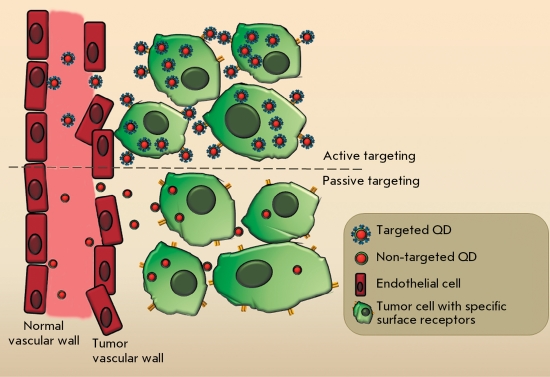
. Schematic illustration of passive and active tumor-targeting after system administration of QD.

Since a vast number of significant oncomarkers are represented by proteins which have regulatory functions in normal cells and which participate in signal transduction, study of the functioning of these proteins is important for understanding the nature and mechanisms of the malignization process. QDs conjugated with an epidermal growth factor (EGF) were used to study the mechanisms of EGF internalization and signal transduction pathways with the participation of proteins from the erbB1/2/3 family of transmembrane tyrokinase receptors [11].

It was demonstrated that QDs can be used to study the motility of tumor cells with the purpose of determining their invasive potential [77]. Use of QDs as markers for imaging of the trajectory of cell motion is less laborious and allows to obtain more reliable data as compared with the conventional Boyden’s chamber assay.

 These novel and extremely interesting directions of study have as yet not found application in clinical diagnostics; however, they will undoubtedly be developed as a domain of fundamental science and will help to obtain new knowledge on tumor pathogenesis. 

## 4. *In vivo* animal imaging

 During the past five years, considerable progress has been made in the application of QDs as fluorophores in experiments on cells and fixed tissues. Meanwhile, the use of these nanoparticles for imaging in multicellular organisms, especially in such highly organized ones as mammals, is only in the early stages of development. 

 Two major problems emerge during fluorescent labeling at the whole-body level: 1) signal attenuation due to the increased size of the organism and tissue thickness, and 2) the difficulty of delivering fluorophores to the target cells and tissues. 

A significant obstacle is the depth of fluorescence penetration, since biological tissues absorb most of the signals that are used for imaging; furthermore, they are characterized by a considerable autofluorescence in the green region of the spectrum. However, in the IR region there exists the so-called “optical window” (650–1300 nm), in which light absorption by living tissues is minimal. The existence of such a window results from the fact that the minimum level of absorption of the major chromophores in mammals (blood, flavins, vitamins, and NAD(P)H) lies in this region [78]. That is why for *in vivo* imaging, QDs fluorescing in the near-IR region (700–800 nm) are used, allowing to improve the brightness of the resulting signal and to reduce the background.

 Fluorophore delivery to target cells in the organism of a mammal is complicated and consists of multiple stages, since the substances being delivered need to penetrate through a number of structural and physiological barriers, including the vascular endothelium, the immune barrier, and the metabolic degradation of the introduced substances. Moreover, after system administration the fluorophore can be delivered via the blood stream into non-target organs and organism tissues and accumulate there, thus reducing the contrast and increasing the possibility of false-positive signals and the manifestation of a toxic effect. Therefore, the excess fluorophore that has not bound to the target cells should be quickly and completely removed from the organism. 

**4.1 Tumor detection using QDs**

In a living organism, accumulation of QDs injected intravenously in tumor tissue for its subsequent imaging is possible through two mechanisms: 1) a passive mechanism, which is typical of particles of a certain size, and 2) an active mechanism, using the targeting agent [53] ( *Fig. 6* ).

In the case of the passive mechanism, nanometer-sized particles accumulate preferentially at tumor site due to its structural features. Such particles can penetrate into a tumor with ease, due to the increased permeability of vascular walls, and remain there as a result of the impaired lymphatic drainage therein. It has been demonstrated that the capillary permeability of the endothelial barrier in newly vascularized tumors is considerably higher than that in normal tissues. Normal blood vessels are lined with a unfenestrated endothelium; hence, the penetration of macromolecules and nanoparticles into the tissue is impeded. Blood vessels formed during the tumor-induced angiogenesis are characterized by a nontypical structure and wide endothelial pores. These pores are so large that molecules up to 400 nm in size can leave the vessels and accumulate in tumor tissue [79]. Moreover, there is almost no lymphatic drainage in tumor tissue; therefore, macromolecules stay there for a considerable amount of time. The described enhanced permeability and retention effect (EPR) is used for the delivery of therapeutic and diagnostic agents based on latexes, liposomes, and other particles into tumors [80]. In the case of the passive delivery, nontargeted PEG-coated QDs that possess a minimal level of nonspecific binding with proteins and blood cells are used [53].

 To provide active delivery of QDs to tumors, they are supplied with targeting molecules capable of binding to the specific receptors exposed on the tumor cell surface (see Section 2.1). 

The possibility of intravital labeling of tumors with quantum dots was first demonstrated on mouse models. It was demonstrated that after intravenous administration, QDs conjugated to peptides specific to various types of tumors and their vessels are selectively accumulated in the tumor vasculature [31].

**Fig. 7:**
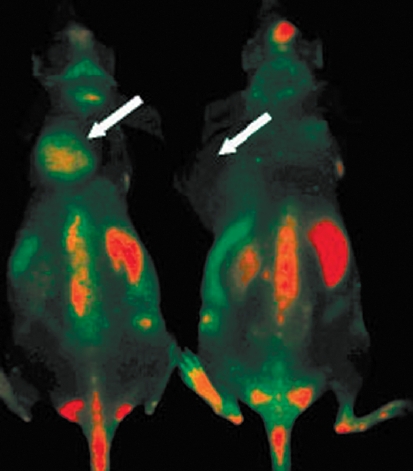
*In vivo* fluorescence imaging of U87MG tumor-bearing mice (left shoulder, indicated by white arrows) injected with targeted (left) and nontargeted QD (right). The mice autofluorescence is color-coded green, while the unmixed QD signal is color-coded red. Prominent uptake in the liver, bone marrow, and lymph nodes was also visible. Reprinted by permission from the American Chemical Society: [Nano Letters] (Cai W, Shin D.W., Chen K, Gheysens O, Cao Q, Wang S.X., Gambhir S.S., Chen X. Nano Lett. 2006 6:669–676), copyright (2006).

The first progress in the *in vivo* application of QDs has stimulated a large number of studies devoted to the intravital imaging of human model tumors in animal organisms using QDs targeted at different tumor markers. Full-size antibodies and their fragments, specific peptides, and natural ligands were used as targeting ligands with equal success ( *Table* ). The results of these studies demonstrate that the use of the mechanism of active targeting, as compared with that of passive targeting, considerably enhances QD accumulation in the tumor regardless of the type of QDs employed and of the type of the targeting agent ( *Fig. 7* ). The use of targeted QDs as fluorophores, in combination with modern optical imaging methods, allows to perform imaging of not only solid tumors, but also metastases in organs [65] and bone tissue [58] and to reveal micrometastases at the early stages of the disease [81]. It should be noted that in all cases, along with successfullabeling of tumors *in vivo* using both the active and passive mechanisms, nonspecific accumulation of QDs in different organs of model animals was observed, primarily, in the liver, spleen, and lymph nodes ( *Fig. 7* ).

In addition, locally introduced QDs can also be of great diagnostic significance. Thus, it was demonstrated that QDs of different colors injected into the peripheral areas of the body were located in different lymph nodes, giving different coloration to them [82]. In recent times, a significant number of studies have been devoted to the imaging of sentinel lymph nodes, along which the metastatic spread typically occurs [83, 84]. Intraoperative imaging of the primary tumor, along with sentinel lymph nodes, provides the possibility of determining the size of the surgery field and the necessity of lymphodissection [85].

The size of QDs and their ability to induce two-photon excitation have prompted researchers to investigate the potential of these particles as promising contrast agents for angiography; possible alternatives to the fluorescent dextrane conventionally used for these purposes. Because of the large cross-section of two-photon absorption (larger than that of conventional organic dyes by 2–3 orders of magnitude), QDs can be excited in the IR range. This fact allows to attain a higher resolution at a great tissue depth (since long waves are less scattered than short waves) and reduce the level of phototoxicity (the exciting photons in the IR region of the spectrum possess a lower energy; therefore, they are less destructive for the tissue under investigation) [86]. Indeed, the use of two-photon excitation of QDs for contrasting the blood vessels of tumor cells considerably enhances the imaging contrast, in comparison with conventional methods [87].

Thus, the results of intravital imaging of tumors presented above demonstrate that, due to the high level of absorption by organs of the reticuloendothelial system (RES) and the absence of complete removal from the organism (see Section 5.2 of this review), the clinical application of QDs as contrast agents for *in vivo* imaging is accompanied by certain difficulties. At the same time, the exceptional brightness, high quantum yield, and large cross section of two-photon absorption, which determines the fluorophore brightness in multiphoton microscopy, allow to successfully utilize QDs as imaging agents for the study of the anatomy and pathophysiology of tumors on animal models. The use of QDs considerably improves the existing methods of intravital microscopy of tumors and their microenvironment. The combination of the exceptional spectral properties of QDs and modern technologies that allow us to obtain *in vivo * images with high resolution may result in a considerable breakthrough in the understanding of tumor biology.

**4.2 QD biodistribution and pharmacokinetics**

The key success factor of *in vivo* diagnostics of tumors are: high fluorophore content in a tumor, in comparison with that found in normal tissues and blood, and the absence of false-positive signals. In addition, it is essential that diagnostic agents be capable of rapid excretion from the organism.

It was demonstrated *in vitro * that the action and biological fate of QDs in the cell depends to a considerable extent on the size and chemical properties of the surface of these particles [88, 89]. The same parameters were assumed to play a significant role in the distribution of QDs in the organism, as well.

When studying the biodistribution of QDs in the organism of model animals, it turned out that all QDs were completely cleared from the bloodstream to accumulate in organs and tissues [90], mostly in RES organs (liver, spleen and lymph nodes). Similar results were obtained in tumor imaging: along with label accumulation in tumor, some QDs also remained in RES organs [25, 31, 32, 53, 65]. Almost in all cases, no QDs were detected in the lung, heart, muscle, or brain tissues; in a number of studies, a small amount of QDs was found in the kidneys. Contrary to expectations, such distribution was independent of surface properties and the type of targeting agent (or the absence of it) conjugated with QD, while the presence of PEG on the particle surface resulted in a slight increase in its blood half-life but did not completely prevent accumulation in these organs. The QD blood half-life varied from several minutes to several tens of hours and depended to a large extent on their hydrodynamic diameter [91], as well as the surface charge and structure [92].

In this context, direct comparison of QDs and the standard organic dye Alexa Fluor 680, both conjugated with anti-IGF1R-antibodies in experiments *in vivo* on model animals, is of interest [55]. It was shown that both fluorophores can allow specific imaging of breast tumor; however, QDs are considerably poorer excreted from the organism, accumulating in RES organs.

The initial results of the investigation of the effect of the size and dimensions of the QD surface on their distribution in the organism after intravenous administration are quite controversial. It was shown in a series of studies that the surface coating [93, 93] and size [94] have a significant effect on the pharmacokinetics and biodistribution of the particles. Conversely, in the systematic study [95] performed in view of all factors that may have an effect on biodistribution, no significant differences were observed for QDs of different sizes, different charges, and those with the presence or absence of different molecules (albumin, PEG) on the surface – all QDs mostly accumulated in the liver and spleen.

QDs can be excreted from the organism via two paths: via the kidneys and the liver [93, 96]. The excretion path to the largest extent depends on two parameters of the particle: their size and surface coating, which determines the tendency towards adsorption of the proteins of the blood serum [78]. The investigation of the excretion from the organism of specially designed series of QDs of different sizes and with different coatings [59] demonstrated that one of the required condition for the full excretion of nanoparticles from the organism via the kidneys is a value of the hydrodynamic diameter of a QD less than 5.5 nm (i.e., below the renal filtration threshold). Currently, all synthesized QDs with fluorescence in red and near-IR ranges that are used for *in vivo* imaging possess a higher size value (approximately 10 nm) and cannot be removed from the organism via the kidneys. Moreover, these QDs are coated with a polymer to improve stability and contain charged functional groups and PEG on their surface, which further increases their size. Thus, the hydrodynamic diameter of popular commercial QDs (Invitrogen) is 15–19 nm [82]. There is only a single excretion path for such non-biodegradable QDs: via the liver, with bile. This process is very slow and inefficient; and the long-term stay of nanoparticles in RES organs increases the possibility of QD shell degradation and the rise of a toxic effect. Thus, although QDs are ideally suitable for *in vivo* imaging of tumors in terms of their parameters, with the exception of their hydrodynamic diameter, their accumulation in the liver, spleen, and other RES organs is inevitable. Interestingly, a number of authors mention the renal accumulation of QDs with a hydrodynamic diameter significantly higher than the renal filtration threshold [95, 97]. In the absence of additional studies, it is difficult to say whether these data are an artifact or attest to some other unstudied mechanisms of interaction between nanoparticles and the living organism. Either way, the complicated excretion from the organism remains one of the major impediments to the use of QDs in the human organism.

##  5. RISKS IN USING QDs IN BIOLOGICAL AND MEDICAL STUDIES 

The unique physicochemical properties of QDs make them extremely attractive fluorophores for the *in vivo* imaging of living objects. The pioneering studies in this field began quite recently (less than 10 years ago); in fact the search for a design of QD optimal for these purposes is ongoing. In this regard, QDs that are used by different laboratories strongly differ in such parameters as their size, shape, charge, concentration, oxidation-reduction properties, surface coating, and physical stability. A wide range of these parameters, in combination with various experimental conditions (treatment time, selection of the model cell lines and media, using the same concentration units, the presence or absence of a targeting agent) make it considerably more difficult to compare the published data on QD biosafety and to get a broad outline. Despite this fact, in a field of extremely diverse and controversial information, a number of regularities have been revealed [90, 98].

**5.1 QD citotoxicity**

The cytotoxic effect of QDs is largely determined by four main factors: the presence of heavy metal ions in their composition, the ability to generate reactive oxygen species (ROS), colloidal instability, and nonspecific interaction with biological molecules [90, 98].

First-generation QDs, consisting only of the fluorescent core (CdTe or CdSe) and stabilized by thiol ligands (e.g., cysteine or MAA), can easily be subjected to oxidation and degradation to release toxic cadmium ions [99] and are capable of inducing ROS formation [100]. Such particles are extremely toxic for culture cells even in small concentration; hence, they are not suitable for investigations on living objects. Second-generation QDs are coated with a shell made of an inert zinc sulfide in order to prevent non-radiative energy dissipation. Moreover, it was found that such a shell actually impedes oxidation and degradation of the fluorescent core and hinders the release of cadmium ions, considerably reducing cytotoxicity. Meanwhile, insufficient colloidal stability is typical of QDs stabilized by an inexpensive and simple method using small thiol ligands [99]. The deposition of such QD aggregates on the cell surface, even without penetration of the cell, may lead to physical damage, functional abnormalities, and as a result, cell death [89]. In principle, second-generation QDs can be used for short-term investigations on cell cultures; however, a significant risk exists when they are used in organisms.

Today, third-generation QDs are used in most biological studies; they are represented by CdSe/ZnS particles coated by a polymeric or silicon shell. These QDs possess a much higher colloidal, chemical, and optical stability as compared with their analogues coated with small ligands. The third-generation QDs manifest some toxicity in cell cultures only under extreme conditions or when used at concentrations that exceed the concentration required for staining and imaging of cell targets by an order of magnitude [89, 101]. These QDs are the most promising for use in the organism. However, it should be taken into consideration when designing them that QDs are not molecules but nanoparticles, and that the physicochemical properties of their surface, rather than their composition, are more important factors in toxicity manifestation (the same is true for other nanoparticles). Some bioinert nanoparticles (gold, carbon) have the same toxic effect on cells as QDs. For example, gold nanoparticles and QDs coated with an amphiphilic polymer shell caused the same physical damages to a mammary cell culture and induced detachment from the substrat [89]. Thus, although the additional, secondary stable shell prevents oxidation and degradation of a QD, it may itself contribute to the overall toxicity of the particles [102].

**Table 2:** Human cancer cells imaging using quantum dots

Tumor type	Cell line (model)	Target/ oncomarker	Targeting module	Quantum dots	Imaging method	Comments	Reference
Prostate cancer	C4-2	PSMA	J591 (full-size monoclonal antibody)	CdSe/ZnS core-shell QD coated amphiphilic polymer	Fluorescent microscopy of cells and tissue sections, whole-body* in vivo *imaging using macro-illumination system	Passive and active QD tumor targeting were compared.	[53]*
	LNCaP (carcinoma)		A10 RNA aptamer	Carboxyl core-shell CdSe/ZnS QD (EviTag)	Confocal microscopy of cells	Authors report the first example of multifunctional nanoparticles ( QD-doxorubicin conjugates) for targeted cancer imaging and therapy.	[35]
	C4-2B (bone metastatic carcinoma)		J591	Qdot® 800 Antibody Conjugation Kit (Invitrogen)	Whole-body* in vivo *imaging using IVIS Imaging System	Targeted QD were used successfully for bone metastases detection.	[58]*
	LNCaP		GPI peptide	Cysteine-coated CdSe/ZnCdS core-shell QD, PEGylated	Fluorescent microscopy of cells, intraoperative fluorescent*post mortem*imaging of internal organs	Small IR QDs for in vivo imaging were created.	[59]*
		α_v_β_3_integrin	RGD peptide				
Breast cancer	MDA-MB-435 (ductal carcinoma)	Endothelium of tumor blood vessels	GFE and LyP-1 peptides	MAA-coated CdSe/ZnS core-shell QD	Confocal microscopy of cells and tissue sections	Authors postulated that QD PEGylation prevents nonselective accumulation of QD in RES.	[31]*
	SKBR-3 (adenocarcinoma)	HER2/neu	Trastuzumab/Herceptin® (humanized monoclonal full-size antibody)	Carboxyl CdSe/ZnS core-shell QD stabilized with amphiphilic polymer (Quantum Dot Corporation)	Fluorescent microscopy of fixed cells	QDs were used to label different types of targets at different subcellular locations and with different types of specimens (cultured live cells, fixed cells, and tissue sections).	[16]
	MCF-7	p-glycoprotein	Anti-р-glycoprotein primary antibody and QD-conjugated anti-mouse polyvalent goat secondary antibody	Cysteine-modified CdSe/ZnS core-shell QD coated with polymer bearing surface amino group	Confocal microscopy of cells, fluorescent IHC	Higher photostability of QD as compared with organic dyes was demonstrated.	[60]
	MDA-MB-435	α_v_β_3_integrin	RGD peptide	Qdot® 705 ITK™ amino (PEG) quantum dots» ( Invitrogen)	Fluorescent microscopy of cells	___	[32]
	MCF-7 (adenocarcinoma а), BT-474 (ductal carcinoma)	EGFR, HER2/neu	Full-size monoclonal antibody	Qdot® Antibody Conjugation Kit (Invitrogen)	Fluorescent microscopy of tissue section	IHC detecting of five tumor markers labeled with QD.	[61]
	KPL-4	HER2/neu	Trastuzumab/Herceptin®	Qdot® Antibody Conjugation Kits (Invitrogen)	*In vivo *fluorescent microscopy	*In vivo*real-time tracking of single QD after i.v. injection.	[54]*
	SK-BR-3, MCF-7/HER2	HER2	scFv antibody fragment	Carboxyl CdSe/ZnS core-shell polymer-coated QD (Invitrogen)	Fluorescent microscopy and flow cytometry of cells,*in vivo*whole-body imaging using macro-illumination system	Creation of multifunctional structures based on immunoliposome and QD.	[62]
	KPL-4	HER2/neu	Trastuzumab/Herceptin®	Qdot® 800 Antibody Conjugation Kit (Invitrogen)	*In vivo *fluorescent microscopy	In vivo real-time tracking of single QD after i.v. injection.	[63]*
	MCF-7	IGF1R	AVE-1642 (humanized monoclonal antibody)	Qdot® Antibody Conjugation Kits (Invitrogen)	Fluorescent microscopy, flow cytometry	Detection of expression level of cell surface receptor.	[55]
Liver cancer	HCCLM6 (hepatocellular carcinoma)	AFP	Mouse anti-human AFP monoclonal antibody	CdSe/ZnS core-shell QD modified thioglycolic acid	*In vivo*spectroscopy imaging and*post mortem*confocal microscopy of tumor	Passive and active QD tumor targeting were compared.	[64]*
			Mouse anti-human AFP monoclonal antibody	CdSe/ZnS core-shell QD modified thioglycolic acid	Whole-body in vivo imaging, serum biochemical examination, confocal microscopy of tissue sections, tissue distribution of Cd and Se using`ICP-MS quantitative assessment	*In vivo*visualization of tumor and metastases. Cytotoxicity*in vitro*, the acute toxicity*in vivo*, the hemodynamics and quantitative tissue distribution were estimated.	[74]*
Pancreatic cancer	MIA PaCa-2 (carcinoma)	Claudin-4, PSCA	Full-size monoclonal antibody	InP/ZnS core-shell QD modified mercaptosuccinic acid	Whole-body imaging using Maestro macro-illumination system, confocal microscopy of tissue sections	Imaging by non-cadmium-based nontoxic QD	[56]
	MIA PaCa-2	EGFR	scFv antibody fragment	CdSe/ZnS core-shell QD coated amphiphilic polymer with PEG	Confocal microscopy of tissue sections	Tissue distribution of targeted and non-targeted QD was investigated	[25]*
Colorectal cancer	HCT166	EGFR	EGF	Qdot® 800 ITK™ amino (PEG) quantum dots (Invitrogen)	Whole-body*in vivo*imaging using IVIS system,*ex vivo*fluorescent imaging of organs	Three distinct phases of tumor influx, clearance and equilibration of accumulation of targeted and non-targeted QD were observed	[66] *
Cervical cancer	HeLa (adenocаr­cinoma)	p-glycoprotein	4Е3 (full-size monoclonal antibody)	DHLA- modified CdSe/ZnS core-shell QD	Fluorescent microscopy of live nonfixed cells	Long-time preservation of QD fluorescence were demonstrated	[67]
Brain-growth	U-87 MG (glioblastoma)	α_v_β_3_integrin	RGD peptide	Qdot® 705 ITK™ amino (PEG) quantum dots (Invitrogen)	Confocal microscopy of cells and tissue sections. Whole-body*in vivo*imaging using Maestro macro-illuminating system.	Passive and active QD tumor targeting were compared	[32]*
Tongue cancer	Tca8113 (squamous cell carcinoma)	p- glycoprotein	Anti-р-glycoprotein primary antibody, biotinylated anti-mouse polyclonal secondary antibody, streptavidin, biotin-conjugated QD	CdTe core QD modified with thioglycolic acid	Fluorescent microscopy	Imaging using water-synthesized QD	[57]
Ovarium cancer	А431 (squamous cell carcinoma)	EGFR	EGF	QD-streptavidin conjugates (Invitrogen)	Confocal microscopy and flow cytometry of cells	Single-molecule fluorescent imaging using QD	[11]
					Fluorescent microscopy, FRET, AFM		[68]
Naso­pharyn­geal cancer	KB (squamous cell carcinoma)	Folate receptor	Folic acid	InP/ZnS	Confocal and two-photon microscopy	Targeted imaging by non-cadmium-based nontoxic QD	[69]

* - whole-body *in vivo* imaging

In summary, it should be noted that since the appearance of the first colloidal QDs for biological applications, significant work has been carried out to reduce their toxicity, mainly by using various shells, and the groundwork has been laid for their *in vivo* application.

**5.2**

*In vivo* **toxicity**

*In vitro* investigation of cytotoxicity is an important and necessary stage in the design of QD-based agents for diagnostics and therapy, since it allows accelerating and standartizing the process of selection of particles for *in vivo* application . However, these studies are usually insufficient for QD use in clinical practice. When using QDs for whole-body imaging, it is necessary to take into account not only the colloidal nature and physicochemical properties of the surface of these particles, but also their interaction with the immune system and the possibility of physicochemical damage occurring under aggressive conditions in the organism, with the release of toxic elements from their fluorescent core.

To this point, data on the interaction between the immune system and certain types of nanoparticles (liposome, carbon, gold, magnetic) have been obtained [103]. After intravenous administration, nanoparticles were shown to become rapidly subjected to opsonization and subsequent phagocytosis by the cells of the immune system. Moreover, their injection into the blood may result in thrombocyte aggregation, activation of the complement system, and stimulation or suppression of the immune system [103]. With regard to QDs, there has been very little experimental data collected on this topic. It could be assumed that the interaction between QDs and the immune system cells is similar to the interaction that is typical of other nanoparticles. Indeed, it was shown by Japanese researchers [104] that QDs both *in vitro* and *in vivo * do not induce an increase in cytokine production by CD4 ^+ ^ Т lymphocytes, but that they stimulate cell proliferation in the immune system.

The data obtained in the first systematic study of the toxicity and biodistribution of QDs in the organism were published quite recently [95]. Short-term (up to 7 days) and long-term (more than 80 days) biological effects of QDs with different polymeric shells were studied in rat models. The standard clinical biochemical and hematological tests were carried out, as well as histological studies of organs. Contrary to expectations, after intravenous administration of QDs containing carboxyl groups, PEG, or bovine serum albumin on their surface (at a total dosage of 60 nmol/animal introduced within a period of four weeks), no pronounced toxicity was observed for either particle variant.

The researchers studying QD biodistribution in the organism and the possibility of tumor imaging *in vivo * havealso repeatedly observed the absence of indicators of acute toxicity in animals, the absence of necroses, and the retention of tissue morphology during experiments [86, 93, 96]. However, the possibility of QD accumulation and degradation in the organism does not exclude their latent toxicity and delayed effect.

QD stability inside RES tissues and organs is the subject of intense debate. Some authors have demonstrated that polymer-coated QDs retain their morphology and fluorescent properties in tissues over a long period of time (up to 4 months) without degradation and following the release of potentially toxic constituent elements of QDs [96, 97]. At the same time, the degradation of such particles in the organism, which results in fluorescence variation, was observed in some cases [53]. This process appears quite real, since it was shown in experiments *in vitro* that some ROS sources, such as oxygen peroxide and hypochlorous acid, always present in the cell in small quantities, can pass through the polymeric shell and cause a degradation of the fluorescent core [105]. It is also possible that only a part of QDs is subjected to degradation in the organism, while the other part remains intact; the ratio between these parts is just a matter of time (months or even years) [95].

Thus, no apparent toxicity of QDs in model organisms has been shown. However, the existence of numerous risk factors, insufficiency of data, and the absence of long-term studies prevents us from drawing a final conclusion on whether it is safe to use QDs in the organism. The separate, fragmentary and controversial data on QD toxicity found in studies devoted to the biodistribution of these particles emphasize the necessity of wide investigations that would deal with different systems of organs for an adequate assessment of the risks inthe use of QDs * in vivo* .

##  COnclusions: problems and potentials 

Quantum dots are a relatively new class of compounds with seemingly immense potential for use in various types of tumor diagnostics, from the microplate assay for oncomarker detection to noninvasive *in vivo* imaging of tumors. The unique physicochemical properties of QDs, easily tunable fluorescence spectra, a high quantum yield (particularly, in the IR region), the possibility of excitation over a wide range of wavelengths and narrow emission fluorescence peaks, large section of two-photon absorption, and resistance to photobleaching, make possible a considerable broadening of the capabilities of modern methods of fluorescence imaging and optical diagnostics. These fluorophores allow one to solve problems that are difficult to overcome using conventional dyes; e.g., simultaneous detection of several markers, long-term real-time observation of molecular processes, and taking images of tumors deep in tissues. However, when performing a number of routine tasks, the problems associated with the colloidal nature of QDs outweigh the advantages provided by their optical properties.

The relatively large surface area of QDs that is accessible for chemical modifications, coupled with the possibility of binding to other molecules and particles, allows to prepare various QD-based multimodule constructions that contain particles of different nature (gold, magnet, diamond, liposome, etc.) in addition to the QDs ( *Fig. 3* ) [28, 106] and simultaneously possess targeting, diagnostic, and therapeutic properties [107]. These multifunctional nanodevices are intended for simultaneous delivery of an active agent to the tumor whilst monitoring this process [35, 62]. The further optimization of biocompatible QDs will facilitate the development of such innovative approaches in oncology as image-guided surgery, molecular profiling of tumors, as well as personalized diagnostics and therapy [108].

The success of the realization of the high potential of the QD method and its implementation in *in vivo* diagnostics depends on achieving solutions to the following important problems. The first such problem is the necessity of long-term toxicological studies and careful investigation of the delayed effect of QD introduction. Coatings based on polymeric materials have already been designed. They considerably enhance QD bioinertness and appreciably reduced their toxicity. However, because QDs accumulate in reticuloendothelial system organs, their removal from the organism being very slow, how safe it is to use them in a living organism still requires further study. In light of this, two other tasks can be formulated: firstly, the necessity to design a new generation of QDs that would be rapidly removed from the organism, and secondly the study of the effects of a possible introduction of QDs into the environment, whilst ensuring ecological safety upon wide application. Presumably, solving these tasks will lead to the development of new materials and technologies for constructing fluorescent nanoparticles.
